# Supplementary material for: Metagenomic, metabolomic, and sensorial characteristics of fermented Coffea arabica L. var. Castillo beans inoculated with microbial starter cultures
Source: mSystems. 2025 Dec 12;11(1):e01364-25. doi: 10.1128/msystems.01364-25 (PMC12817937; doi:10.1128/msystems.01364-25)
Supplement: Supplemental material — Supplemental methods, figures, and tables. [file msystems.01364-25-s0001.docx]

**Metagenomic, metabolomic and sensorial characteristics of fermented *Coffea arabica* L. var. Castillo beans inoculated with microbial starter cultures**

Maria A. MADRID-RESTREPO^1,2^*, Ana M. LEÓN-INGA^3^, Aida Esther PEÑUELA-MARTÍNEZ ^4^ , Mónica P. CALA^3^, Alejandro REYES^1,2^#

1. Group in Computational Biology and Microbial Ecology, Department of Biological Sciences, Faculty of Science, Universidad de los Andes, Bogotá, Colombia

2. Max Planck Tandem Group in Computational Biology, Faculty of Science, Universidad de los Andes, Bogotá, Colombia

3. MetCore - Metabolomics Core Facility. Vice-Presidency for Research, Universidad de los Andes, Bogotá, Colombia

4. National Coffee Research Center, Cenicafé, Manizales, Colombia

**Running head**: Inoculated Fermentation of *Coffea arabica*: Multi-Omic Analysis

#Address correspondence to Alejandro Reyes, [a.reyes@uniandes.edu.co](mailto:a.reyes@uniandes.edu.co)

*Present address: Maria A. Madrid-Restrepo, KU Leuven, Leuven, Belgium

**Supplementary Material**

**A. Abbreviations**

**RP-LC-MS-QTOF** Reverse Phase - Liquid Chromatography - Mass Spectrometry -Quadrupole Time-of-Flight

**HILIC-LC-MS-QTOF** Hydrophilic Interaction Liquid Chromatography - Mass Spectrometry - Quadrupole Time-of-Flight

**GC-MS-QTOF** Gas Chromatography - Mass Spectrometry - Quadrupole Time-of-Flight

**GSW** Green Spontaneous Wet

**GIW** Green Inoculated Wet

**RSW** Roasted Spontaneous Wet

**RIW** Roasted Inoculated Wet

**B. Methods**

**Untargeted metabolomic analysis by RP-LC-MS-QTOF**

100 µL of sample extract were analyzed using an Agilent Technologies 1260 Liquid Chromatography system coupled to a Q-TOF 6545 time-of-flight quadrupole mass analyzer with electrospray ionization (Agilent, Santa Clara, California, USA). 1 µL of each sample extract was injected onto a C_18_ column (InfinityLab Poroshell 120 EC-C_18_ (100 x 3.0 mm, 2.7 µm) at 30 °C and with a gradient elution composed by 0.1 % (v/v) formic acid in Milli-Q water (Phase A) and 0.1 % (v/v) formic acid in acetonitrile (Phase B). The gradient started at 2% of Phase B, increased for 17 min to 98% B, and was maintained for 2 min to decrease to 2% B and system recondition, with a constant flow rate of 0.4 mL/min. Mass spectrometric detection was performed in positive ESI mode in full scan and iterative MS/MS from 50 to 1100 *m/z* at a rate. Throughout the analysis, two reference masses were used for mass correction: *m/z* 121.0509 [C_5_H_4_N_4_]^+^ and *m/z* 922.0098 [C_18_H_18_O_6_N_3_P_3_F_24_]^+^.

**Untargeted metabolomic analysis by HILIC-LC-MS-QTOF**

2 µL of each sample extract were injected onto an AdvanceBio Ms Spent Media column (150 x 2.1 mm, 2.7 µm) at 40 °C and with a gradient elution composed by 10mM ammonium acetate in acetonitrile: Milli-Q water (50:50) (Phase A) and 10 mM ammonium acetate in acetonitrile: Milli-Q water (95:5) (Phase B). Gradient started in 99% of B-phase, decreased for 15 min to 50% B and was maintained for 1 min to increase again at 99% B and system recondition, with a constant flow rate of 0.4 mL/min. Mass spectrometric detection was performed in negative ESI mode in full scan and iterative MS/MS from 50 to 1100 *m/z*. Throughout the analysis, two reference masses were used for mass correction: *m/z* 112.9856 [C_2_H_4_O_2_NF_3_-NH_4_]^-^ and *m/z* 1033.9881 [(C_18_H_18_O_6_N_3_P_3_F_24_+ trifluoroacetic acid)-H]^-^.

**Untargeted metabolomic analysis by GC-MS-QTOF**

An Agilent Technologies 7890B gas chromatograph coupled to an Agilent Technologies GC/Q-TOF 7250 time-of-flight mass selective detector equipped with a split/splitless injection port (250 °C, split ratio 50) and an Agilent Technologies 7693A automatic injector (Agilent, Santa Clara, California, USA) was used for data acquisition. The electronic ionization source was operated at 70 eV. An Agilent Technologies J&W HP-5MS column (30 m, 0.25 mm, 0.25 µm) was used, the carrier gas was helium at a constant flow rate of 0.7 mL/min. The oven temperature was programmed from 60 °C (1 min) @10 °C/min to 325 °C (10 min). The temperature of the transfer line to the detector, source filament and quadrupole were maintained at 280 °C, 230 °C and 150 °C, respectively. Mass spectrometric detection was performed between 50 to 600 *m/z* at a rate of 5 spectra/sec.

**Untargeted metabolomic analysis sample preparation**

For sample preparation, a total of 1 g of the green and roasted coffee beans were flash-frozen with liquid nitrogen and pulverized with a sterile mortar and pestle. The resulting green and roasted coffee powder was analyzed. Metabolite extraction was performed from 50 mg of sample in 1.5 mL of H_2_O:MeOH (75:25), agitated for 15 mins, followed by ultrasound at 53 kHz for 10 mins. Afterwards, the samples were centrifuged at 24328 xg for 10 mins at room temperature (25 °C) and the supernatant was collected for analysis. For GC-MS analysis, a total of 100 µL of sample extract were taken and dried in a SpeedVac for 2 h and 30 min at 35 °C. After, 20 µL of *O*-methoxyamine in pyridine (15 mg/mL) were added. The samples were shaken for 10 mins, then were left in darkness for 16 h to complete methoximation. The process of silylation was performed adding 20 µL of BSTFA with 1% of TMCS, followed by vortexing during 10 min. The samples were incubated at 70 °C for 1 h, then were left at room temperature for 30 min, and finally 210 µL of methyl stearate in heptane were added as an internal standard (10 mg/L).

Quality control (QC) samples were prepared by mixing equal volumes of the metabolic extract of each sample (100 µL). Sample preparation was performed following the procedure for each analytical platform. QC samples were injected to equilibrate the analytical system, and then analyzed every fourth randomly injected sample to determine the reproducibility of the sample preparation and the stability of each platform.

**Data processing, statistical analysis, and metabolite annotation**

The raw data from the liquid chromatography analyses were exported from Agilent MassHunter Profinder 10.0 software, analyzed and inspected manually using the Recursive Molecular Extraction algorithm. For the gas chromatography, data deconvolution and alignment were performed using Agilent MassHunter Unknowns Analysis B.10.00 and Agilent MassProfiler Professional software, respectively. The processed data were exported to Agilent MassHunter Quantitative software for integration. Data obtained from pre-processing was filtered for reproducibility and presence. For reproducibility filtering, the coefficient of variation (CV) of the area in the quality control (QC) samples was calculated and molecular characteristics with a CV > 20% for liquid chromatography and CV > 30% for gas chromatography were discarded.  For filtering by presence, only data with 100% absence or presence in at least one group were retained.

An unsupervised principal component analysis (PCA) was performed to verify the reproducibility of the analytical platforms and the dispersal of the analyzed samples. For the selection of statistically significant molecular characteristics, comparisons between green coffee beans and roasted coffee beans from inoculated and spontaneous treatments, were evaluated by univariate (UVA) and multivariate (MVA) statistical analysis using MetaboAnalyst 5.0 server. Supervised PLS-DA models were performed to select the molecular characteristics responsible for the separation between groups with the following requirements: 1) UVA: *p-*value < 0.05 or 2) MVA: Variance important in projection (VIP) > 1 with Jack-knife confidence interval (JK) not including 0 for GC-MS, while for LC-MS the conditions were: 1) UVA: *p-*value *< 0.05* and 2) MVA: VIP > 1 with Jack-knife confidence interval (JK) not including 0.

Signals with a significant statistical variation were selected for annotation. Metabolite identification for RP-LC-MS-QTOF was performed considering isotopic mass, isotopic distribution, adduct formation, molecular formula, retention time (RT) and MS/MS spectra. An initial search in online databases Human Metabolome Database (Wishart et al 2022), KEGG (http://genome.jp/keg), MassBank (https://massbank.eu/MassBank/), Lipid MAPS (http://lipidmaps.org), and METLIN (http://metlin.scripps.edu) were conducted for the annotation in the CEU Mass Mediator tool. Formulas for statistically significant features were generated and confirmed using Agilent MassHunter Qualitative Software 10 by autoMS/MS algorithm for QC samples and comparison with information and spectra standards. The confidence levels for the annotation were reported according to the guidelines presented in previous studies (Blaženović et al. 2018). While metabolite identification for GCMS was performed employing Fiehn libraries version 2013, PCDL Manager B.08.00 and NIST (National Institute of Standards and Technology, library 2.2 version 2017). The Human Metabolome Database (Wishart et al 2022), FooDB (https://foodb.ca/), and The Good Scents Company (http://www.thegoodscentscompany.com/) databases were used to identify the organoleptic characteristics and/or biological origin of the annotated metabolites.

**C. Figures**

**
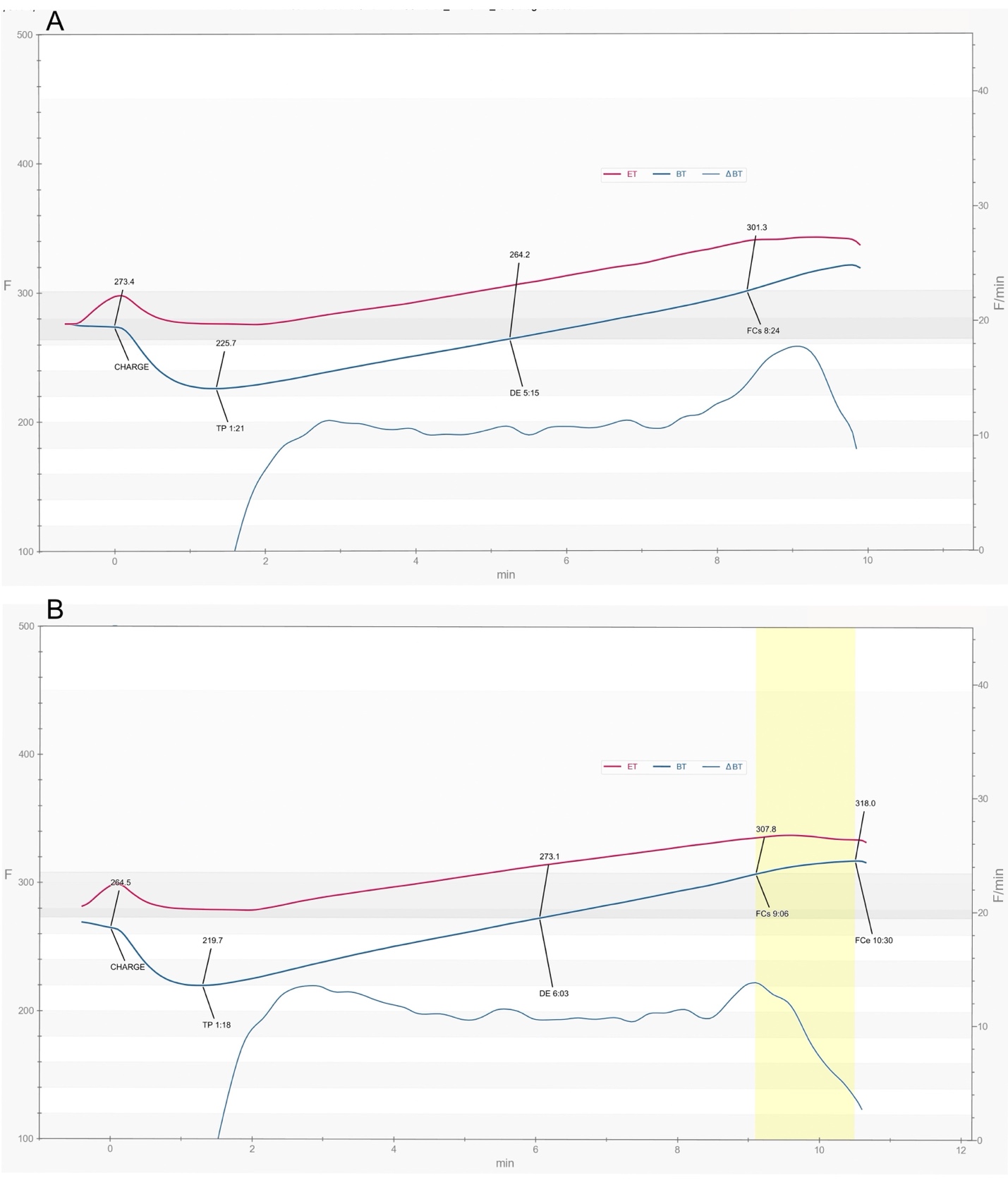
**

**Supplementary Figure 1.** Roasting curves for both spontaneous (**A**) and inoculated (**B**) wet fermented green coffee beans. Coffee roasting is a process where green coffee beans are heated above 200°F to develop their flavor, aroma, and color. During roasting, the “first crack” marks the initial expansion of beans and release of moisture, while “second crack” results in further caramelization, creating a more intense and darker roast flavor. Roasting curves are tailored for each batch to accentuate unique flavor profiles.

**
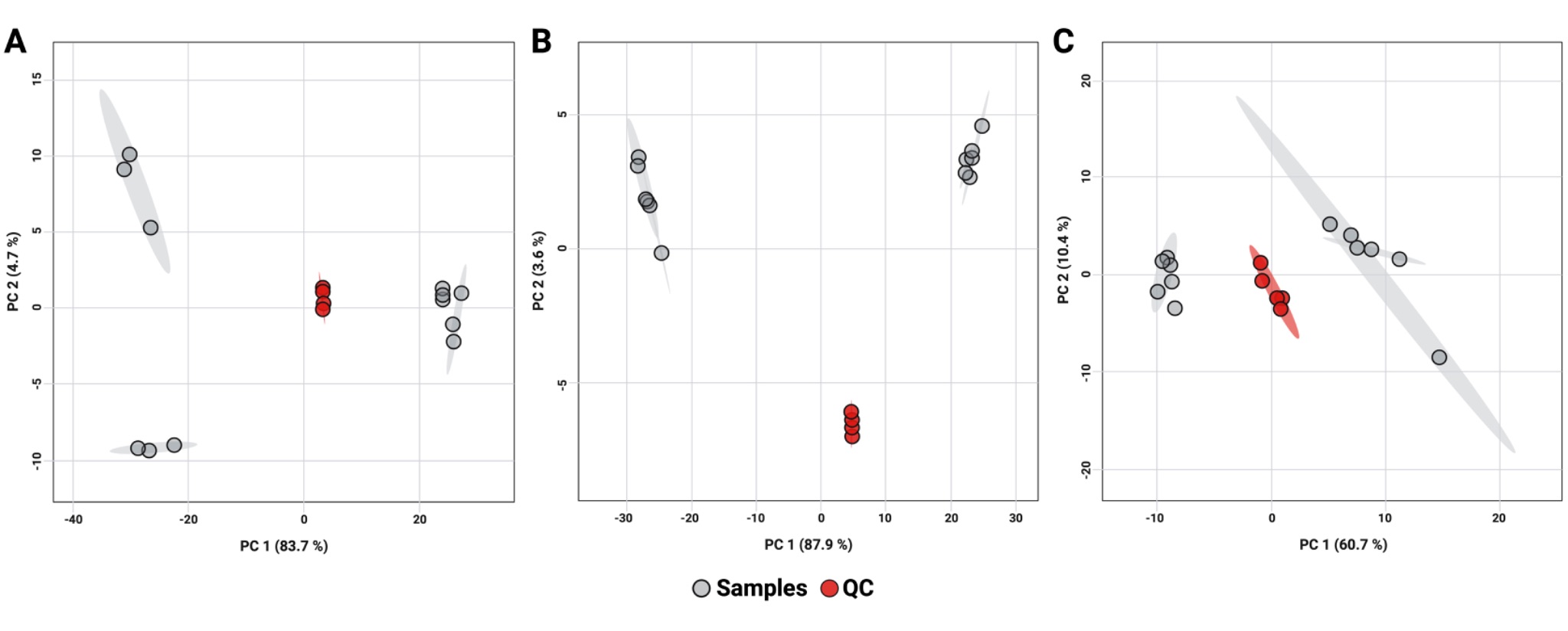
**

**Supplementary Figure 2.** Unsupervised principal component analysis (PCA) for the verification of the reproducibility of the analytical platforms and the dispersal of the analyzed samples.

**
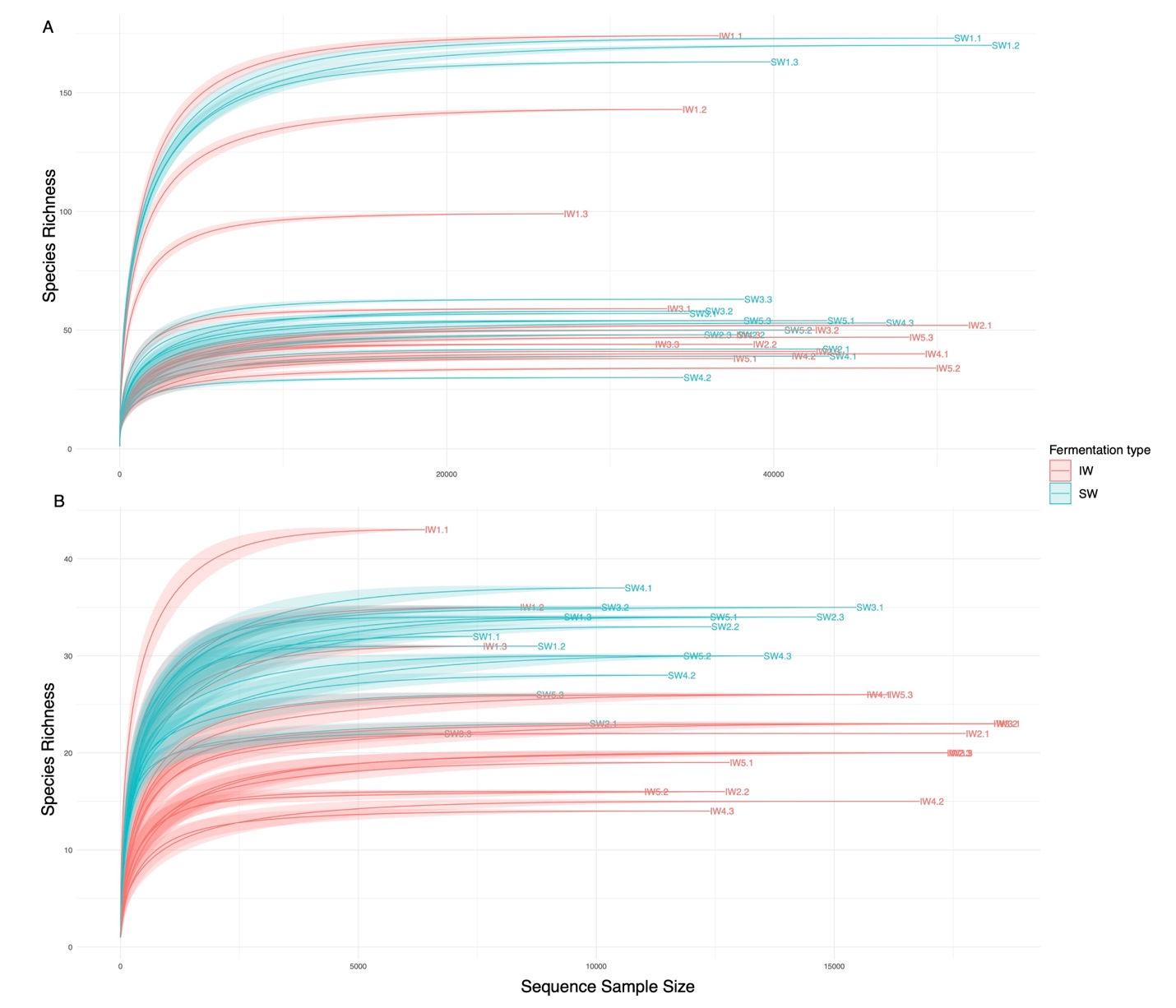
**

**Supplementary Figure 3.** Rarefaction curves for bacterial (**A**) and fungal (**B**) samples.

**D. Tables**

**Supplementary Table 1.** Q-Grader scores associated with (**A**) spontaneous and (**B**) inoculated fermentation from each one of the three cuppers.

**A**. Spontaneous wet fermentation brewed coffee

| Q-grader | Aroma | Flavor | Residual flavor | Acidity | Body | Uniformity | Balance | Clean cup | Sweetness | General |
| --- | --- | --- | --- | --- | --- | --- | --- | --- | --- | --- |
| 1 | 7.75 | 7.75 | 7.75 | 7.75 | 7.75 | 8 | 7.5 | 8 | 8 | 7.5 |
| 2 | 7.75 | 8 | 7.5 | 7.75 | 7.75 | 8 | 8 | 8 | 8 | 7.75 |
| 3 | 8 | 7.75 | 8 | 8 | 8 | 8 | 8 | 8 | 10 | 7.75 |

**B**. Inoculated wet fermentation brewed coffee

| Q-grader | Aroma | Flavor | Residual flavor | Acidity | Body | Uniformity | Balance | Clean cup | Sweetness | General |
| --- | --- | --- | --- | --- | --- | --- | --- | --- | --- | --- |
| 1 | 7.5 | 7.75 | 7.25 | 7.5 | 7.5 | 10 | 7.5 | 10 | 10 | 7.75 |
| 2 | 7.75 | 7.75 | 7.5 | 7.75 | 7.75 | 10 | 7.75 | 10 | 10 | 7.75 |
| 3 | 7.5 | 7.75 | 7.5 | 7.75 | 8 | 10 | 7.5 | 10 | 10 | 7.75 |

**Supplementary Table 2.** Significantly differential compounds from green coffee beans, based on VIP and/or a *p*-value, depending on the analytical platform. Additional information includes compound name, molecular formula, molecular weight, retention time (RT), and ID-level.

| ***Compound name*** | ***Molecular Formula*** | ***Molecular Weight (DB) g/mol*** | ***RT (min)*** | ***mz Error (ppm)*** | ***Analytical platform*** | ***DET*** | ***ID level*** | ***CV in QC (%)*** | ***Adduct*** | ***GIW vs GSW*** | | |
| --- | --- | --- | --- | --- | --- | --- | --- | --- | --- | --- | --- | --- |
|  |  |  |  |  |  |  |  |  |  | **Fold Change** | **P Value** | **VIP** |
| ***Amines*** | | | | | | | | | | | | |
| Ethanolamine | C_2_H_7_NO | 61,0528 | 9,846 | - | GC-QTOF-MS | ESI+ | 1 | 9,75 | - | 1,2 | - | 1,120 |
| Butane-1,4-diamine | C_4_H_12_N_2_ | 88,1000 | 15,733 | - | GC-QTOF-MS | ESI+ | 1 | 11,66 | - | 1,3 | - | 1,113 |
| ***Amino acids, peptides, and analogues*** | | | | | | | | | | | | |
| Alanine | C_3_H_7_NO_2_ | 89,0477 | 12,001 | - | GC-QTOF-MS | ESI+ | 1 | 10,81 | - | 1,3 | - | 1,229 |
| Glycine | C_2_H_5_NO_2_ | 75,0320 | 10,413 | - | GC-QTOF-MS | ESI+ | 1 | 9,14 | - | 0,8 | - | 1,328 |
| Valine | C_5_H_11_NO_2_ | 117,0790 | 7,168 | - | GC-QTOF-MS | ESI+ | 1 | 17,53 | - | 0,8 | 0,048 | 1,344 |
| Tripeptide 1 | C_17_H_22_N_4_O_7_ | 394,1488 | 4,020 | 7 | HILIC-LC-QTOF-MS | ESI- | 3 | 1,46 | [M-H]^-^ | 1,3 | 0,005 | 1,523 |
| Tripeptide 5 | C_14_H_24_N_4_O_6_ | 344,1696 | 5,760 | 0 | HILIC-LC-QTOF-MS | ESI- | 4 | 3,92 | [M-H+CH3COO]^-^ | 1,6 | 0,005 | 1,520 |
| Tripeptide 10 | C_13_H_24_N_4_O_5_S | 348,1467 | 13,580 | 7 | HILIC-LC-QTOF-MS | ESI- | 4 | 4,32 | [M+Cl]^-^ | 0,9 | 0,017 | 1,444 |
| Tripeptide 12 | C_16_H_26_N_6_O_5_ | 382,1965 | 14,150 | 5 | HILIC-LC-QTOF-MS | ESI- | 3 | 2,28 | [M-H]^-^ | 0,7 | 0,020 | 1,429 |
| Tripeptide 13 | C_23_H_26_N_4_O_4_S | 454,1675 | 4,680 | 0 | RP-LC-QTOF-MS | ESI+ | 4 | 9,32 | [M+H-H2O]^+^ | 1,6 | 0,003 | 1,443 |
| ***Benzene and substituted derivatives*** | | | | | | | | | | | | |
| 2-Phenylethanol | C_8_H_10_O | 122,0732 | 9,238 | - | GC-QTOF-MS | ESI+ | 1 | 8,83 | - | 0,7 | 0,018 | 1,464 |
| Benzyl alcohol | C_7_H_8_O | 108,0575 | 8,178 | - | GC-QTOF-MS | ESI+ | 1 | 9,38 | - | 0,0 | 0.031ꝉ | 1,616 |
| Catechol | C_6_H_6_O_2_ | 110,0368 | 10,555 | - | GC-QTOF-MS | ESI+ | 1 | 9,32 | - | 0,2 | 0,014 | 1,489 |
| Phenyllactic acid | C_9_H_10_O_3_ | 166,0630 | 13,978 | - | GC-QTOF-MS | ESI+ | 1 | 13,55 | - | 0,4 | 0,012 | 1,503 |
| Vanillin // Ortho-Hydroxyphenylacetic acid // Ethyl furanyl diketone | C_8_H_8_O_3_ | 152,0473 | 7,030 | 4 | RP-LC-QTOF-MS | ESI+ | 3 | 1,64 | [M+H-H2O]^+^ | 1,3 | 0,004 | 1,431 |
| Verimol G | C_20_H_26_O_5_ | 346,1780 | 15,110 | 4 | RP-LC-QTOF-MS | ESI+ | 3 | 10,78 | [M+H]^+^ | 1,4 | 0,003 | 1,447 |
| ***Carbohydrates and carbohydrate conjugates*** | | | | | | | | | | | | |
| Arabinose | C_5_H_10_O_5_ | 150,0528 | 15,023 | - | GC-QTOF-MS | ESI+ | 1 | 11,72 | - | 0,7 | 0,034 | 1,394 |
| Fructose | C_6_H_12_O_6_ | 180,0634 | 17,325 | - | GC-QTOF-MS | ESI+ | 1 | 11,60 | - | 1,2 | - | 1,123 |
| Glucosamine phosphate | C_6_H_14_NO_8_P | 259,0457 | 16,252 | - | GC-QTOF-MS | ESI+ | 1 | 10,91 | - | 0,6 | 0,018 | 1,463 |
| Mannitol // Sorbitol | C_6_H_14_O_6_ | 182,0790 | 17.928 // 8.510 | - | GC-QTOF-MS // HILIC-LC-QTOF-MS | ESI± | 1 | 8,29 | [M-H]^-^ | 1,9 | 0,015 | 1,480 |
| Methyl galactoside | C_7_H_14_O_6_ | 194,0790 | 20,289 | - | GC-QTOF-MS | ESI+ | 1 | 7,36 | - | 0,4 | 0.019ꝉ | 1,631 |
| Methyl naphthalenetriol xylosylglucoside | C_22_H_28_O_12_ | 484,1581 | 9,990 | 3 | RP-LC-QTOF-MS | ESI+ | 3 | 6,68 | [M+H]^+^ | 0,2 | 0,006 | 1,418 |
| Sucrose | C_12_H_22_O_11_ | 342,1162 | 24,126 | - | GC-QTOF-MS | ESI+ | 1 | 18,07 | - | 1,5 | - | 1,012 |
| ***Carboxylic acids and derivatives*** | | | | | | | | | | | | |
| Fumonisin A1 | C_36_H_61_NO_16_ | 763,3990 | 17,080 | 3 | RP-LC-QTOF-MS | ESI+ | 3 | 3,75 | [M+H-H2O]^+^ | 1,7 | 0,016 | 1,355 |
| Fumonisin B4 | C_34_H_59_NO_13_ | 689,3986 | 18,830 | 7 | RP-LC-QTOF-MS | ESI+ | 3 | 3,04 | [M+Na]^+^ | 1,9 | 0,002 | 1,455 |
| Ginsenoyne F | C_19_H_24_O_3_ | 300,1725 | 18,110 | 4 | RP-LC-QTOF-MS | ESI+ | 3 | 7,52 | [M+H]^+^ | 1,4 | 0,030 | 1,295 |
| ***Cinnamic acids and derivatives*** | | | | | | | | | | | | |
| Feruloylquinic acid | C_17_H_20_O_9_ | 368,1107 | 11,800 | 5 | RP-LC-QTOF-MS | ESI+ | 3 | 19,66 | [M+Na]^+^ | 0,0 | 0,026 | 1,310 |
| Feruloylquinolactone | C_17_H_18_O_8_ | 350,1002 | 1,760 | 2 | HILIC-LC-QTOF-MS | ESI- | 3 | 0,15 | [M-H]^-^ | 0,7 | 0,004 | 1,533 |
| ***Quinic acids and derivatives*** | | | | | | | | | | | | |
| Coumaroylquinic acid | C_16_H_18_O_8_ | 338,1002 | 10.130 // 4.810 | 4 | RP-LC-QTOF-MS // HILIC-LC-QTOF-MS | ESI± | 3 | 2,09 | [M+H]^+^ // [M-H]^-^ | 1,2 | 0,032 | 1,296 |
| 3,4-Dicaffeoylquinic acid | C_25_H_24_O_12_ | 516,1268 | 13,59 | 4 | RP-LC-QTOF-MS | ESI+ | 1 | 1,93 | [M+H-H2O]^+^ // [M+H]^+^ | 1,3 | 0,008 | 1,403 |
| Quinic acid | C_7_H_12_O_6_ | 192,0634 | 17,183 | - | GC-QTOF-MS | ESI+ | 1 | 7,68 | - | 1,2 | - | 1,290 |
| ***Organic acids and derivatives*** | | | | | | | | | | | | |
| 2-ketobutyric acid | C_4_H_6_O_3_ | 102,0317 | 7,426 | - | GC-QTOF-MS | ESI+ | 1 | 25,06 | - | 0,4 | 0,014 | 1,489 |
| 3-Hydroxybutyric acid | C_4_H_8_O_3_ | 104,0473 | 8,279 | - | GC-QTOF-MS | ESI+ | 1 | 5,82 | - | 0,3 | 0,003 | 1,581 |
| Glycolic acid | C_2_H_4_O_3_ | 76,0160 | 6,999 | - | GC-QTOF-MS | ESI+ | 1 | 7,74 | - | 0,8 | - | 1,313 |
| Hydroxyglutaric acid | C_5_H_8_O_5_ | 148,0372 | 13,844 | - | GC-QTOF-MS | ESI+ | 1 | 7,84 | - | 1,4 | 0,032 | 1,403 |
| Isopropyl formate | C_4_H_8_O_2_ | 88,0524 | 4,340 | 3 | HILIC-LC-QTOF-MS | ESI- | 3 | 1,78 | [M-H]^-^ | 0,0 | 0,001 | 1,583 |
| Malic acid | C_4_H_6_O_5_ | 134,0215 | 12,810 | - | GC-QTOF-MS | ESI+ | 1 | 11,44 | - | 1,3 | 0,032 | 1,403 |
| Oxalic acid | C_2_H_2_O_4_ | 89,9953 | 8,005 | - | GC-QTOF-MS | ESI+ | 1 | 13,18 | - | 1,3 | - | 1,100 |
| Pyruvic acid | C_3_H_4_O_3_ | 88,0160 | 6,622 | - | GC-QTOF-MS | ESI+ | 1 | 5,88 | - | 0,7 | 0,043 | 1,360 |
| ***Fatty Acyls*** | | | | | | | | | | | | |
| Cibaric acid | C_18_H_28_O_5_ | 324,1937 | 18,160 | 9 | RP-LC-QTOF-MS | ESI+ | 3 | 6,88 | [M+Na]^+^ | 1,4 | 0,012 | 1,377 |
| ***Glycerophospholipids*** | | | | | | | | | | | | |
| PC 18:1 | C_26_H_52_NO_7_P | 521,3481 | 19,230 | 5 | RP-LC-QTOF-MS | ESI+ | 3 | 2,92 | [M+H]^+^ | 0,5 | 0,020 | 1,340 |
| PI 37:7 | C_46_H_75_O_13_P | 866,4945 | 5,210 | 7 | HILIC-LC-QTOF-MS | ESI- | 4 | 3,27 | [M-H]^-^ | 1,5 | 0,048 | 1,321 |
| ***Organoheterocyclic compounds*** | | | | | | | | | | | | |
| Cafestol | C_20_H_28_O_3_ | 316,2038 | 11,030 | 4 | RP-LC-QTOF-MS | ESI+ | 2 | 9,04 | [M+H]^+^ | 1,4 | 0,046 | 1,240 |
| Caffeine | C_8_H_10_N_4_O_2_ | 194,0804 | 16,885 | - | GC-QTOF-MS | ESI+ | 1 | 7,43 | - | 1,1 | - | 1,196 |
| Furfuryl acetate | C_7_H_8_O_3_ | 140,0473 | 6,680 | 5 | RP-LC-QTOF-MS | ESI+ | 2 | 4,04 | [M+H]^+^ | 0,0 | 0.046ꝉ | 1,481 |
| Niacin | C_6_H_5_NO_2_ | 123,0320 | 10,162 | - | GC-QTOF-MS | ESI+ | 1 | 20,89 | - | 1,3 | - | 1,108 |
| Riboflavin | C_17_H_20_N_4_O_6_ | 376,1383 | 16,501 | - | GC-QTOF-MS | ESI+ | 1 | 15,24 | - | 0,8 | - | 1,010 |
| ***Organooxygen compounds*** | | | | | | | | | | | | |
| 1,3-propanediol | C_3_H_8_O_2_ | 76,0524 | 6,714 | - | GC-QTOF-MS | ESI+ | 1 | 8,00 | - | 2,7 | 0,002 | 1,593 |
| 2,3-butanediol | C_4_H_10_O_2_ | 90,0681 | 6,651 | - | GC-QTOF-MS | ESI+ | 1 | 5,65 | - | 0,8 | - | 1,194 |
| Acetol | C_3_H_6_O_2_ | 74,0368 | 15,076 | - | GC-QTOF-MS | ESI+ | 1 | 18,09 | - | 0,5 | 0,009 | 1,518 |
| Furancarboxaldehyde | C_5_H_4_O_2_ | 96,0211 | 1,890 | 4 | HILIC-LC-QTOF-MS | ESI- | 3 | 0,84 | [M-H]^-^ | 0,9 | 0,040 | 1,348 |
| Myo-Inositol | C_6_H_12_O_6_ | 180,0634 | 19,460 | - | GC-QTOF-MS | ESI+ | 1 | 5,49 | - | 1,3 | - | 1,227 |
| ***Others*** | | | | | | | | | | | | |
| Mercaptomethyl butanol // Methylthiobutanol | C_5_H_12_OS | 120,0609 | 14,330 | 4 | HILIC-LC-QTOF-MS | ESI- | 3 | 4,80 | [M-H]^-^ | 1,6 | 0,013 | 1,467 |
| ***Phenylpropanoids and polyketides*** | | | | | | | | | | | | |
| Afzelechin O-rhamnopyranoside | C_21_H_24_O_9_ | 420,1420 | 15,910 | 4 | RP-LC-QTOF-MS | ESI+ | 3 | 8,78 | [M+H]^+^ | 1,4 | 0,013 | 1,368 |
| Ethyl vanillin //Antiarol | C_9_H_10_O_3_ // C_9_H_12_O_4_ | 166.0630 // 184.0736 | 5,630 | 2 // 1 | HILIC-LC-QTOF-MS | ESI- | 3 | 2,03 | [M-H]^-^ // [M-H-H2O]^-^ | 0,4 | 0,020 | 1,427 |
| ***Prenol Lipids*** | | | | | | | | | | | | |
| Cofaryloside | C_26_H_42_O_10_ | 514,2778 | 5,220 | 4 | HILIC-LC-QTOF-MS | ESI- | 3 | 5,07 | [M-H]^-^ | 1,2 | 0,026 | 1,402 |
| Dihydroxysugiol | C_20_H_28_O_4_ | 332,1988 | 19,260 | 2 | RP-LC-QTOF-MS | ESI+ | 3 | 6,00 | [M+H]^+^ | 0,4 | 0,031 | 1,298 |
| ***Purines and purine derivatives*** | | | | | | | | | | | | |
| Adenosine // Deoxyguanosine | C_10_H_13_N_5_O_4_ | 267,0968 | 5,250 | 3 | HILIC-LC-QTOF-MS | ESI- | 3 | 0,93 | [M-H]^-^ | 0,6 | 0,010 | 1,482 |
| ***Pyridines and derivatives*** | | | | | | | | | | | | |
| Ethyl pyridine | C_7_H_9_N | 107,0735 | 1,620 | 4 | RP-LC-QTOF-MS | ESI+ | 2 | 6,92 | [M+H]^+^ | 0,6 | 0,011 | 1,390 |

^a^CV for QC, coefficient of variation in the metabolites in the QC samples; ^b^p-value corresponding to the p-values calculated by Tukey pairwise comparisons (p-value < 0.05) and ꝉ corresponding to the p-values calculated by the Benjamini–Hochberg false discovery rate post hoc correction (FDR < 0.05); ^c^VIP, variable importance in projection; Fold Change, indicate the change in the abundance of the specified comparison calculated as (average case/average control), where values greater than 1 indicated an increase while values less than 1 indicate a decrease in the case group; RT: Retention time; RP-LC-QTOF-MS: liquid chromatography-quadrupole time-of-flight mass spectrometry; HILIC-LC-MS-QTOF: Hydrophilic Interaction Chromatography system coupled to a Q-TOF Mass Spectrometer; GC-QTOF-MS: Gas Chromatography coupled to a Q-TOF Mass Spectrometer. The ID levels show confidence in the identification and were assigned from 0 to 4, where: 0 = unambiguous 3D structure; 1 = confident 2D structure or reference standard match; 2 = probable structure or coincidence with literature spectra; 3 = possible structure, confirmation with molecular formula; and 4 = Unknown feature or only match with libraries.

**Supplementary Table 3.** Significantly differential compounds from roasted coffee beans, based on VIP and/or a *p*-value, depending on the analytical platform. Additional information includes compound name, molecular formula, molecular weight, retention time (RT), and ID-level.

| ***Compound name*** | ***Molecular Formula*** | ***Molecular Weight (DB) g/mol*** | ***RT (min)*** | ***mz Error (ppm)*** | ***Analytical platform*** | ***DET*** | ***ID level*** | ***CV in QC (%)*** | ***Adduct*** | ***RIW vs RSW*** | | |
| --- | --- | --- | --- | --- | --- | --- | --- | --- | --- | --- | --- | --- |
|  |  |  |  |  |  |  |  |  |  | **Fold Change** | **P Value** | **VIP** |
| ***Amines*** | | | | | | | | | | | | |
| Butane-1,4-diamine | C_4_H_12_N_2_ | 88,1000 | 15,733 | - | GC-QTOF-MS | ESI+ | 1 | 11,66 | - | 1,9 | 0,011 | 1,783 |
| ***Amino acids, peptides, and analogues*** | | | | | | | | | | | | |
| Valine | C_5_H_11_NO_2_ | 117,0790 | 7,168 | - | GC-QTOF-MS | ESI+ | 1 | 17,53 | - | 0,1 | - | 1,574 |
| Dipeptide 1 | C_17_H_26_N_4_O_6_ | 382,1852 | 4,330 | 7 | HILIC-LC-QTOF-MS | ESI- | 3 | 2,37 | [M-H]^-^ | 1,1 | 0,028 | 1,312 |
| Dipeptide 2 | C_11_H_20_N_2_O_5_ | 260,1372 | 10,530 | 0 | HILIC-LC-QTOF-MS | ESI- | 3 | 13,32 | [M-H-H2O]^-^ | 0,7 | 0,027 | 1,316 |
| Tripeptide 2 | C_15_H_30_N_4_O_4_ | 330,2267 | 4,310 | 0 | HILIC-LC-QTOF-MS | ESI- | 4 | 2,54 | [M+Cl]^-^ | 0,8 | 0,025 | 1,323 |
| Tripeptide 3 | C_17_H_24_N_4_O_6_ | 380,1696 | 4,660 | 7 | HILIC-LC-QTOF-MS | ESI- | 3 | 1,51 | [M-H]^-^ | 1,1 | 0.018ꝉ | 1,478 |
| Tripeptide 4 | C_13_H_22_N_4_O_7_ | 346,1488 | 5,310 | 1 | HILIC-LC-QTOF-MS | ESI- | 4 | 3,80 | [M+Cl]^-^ | 0,7 | 0.009ꝉ | 1,502 |
| Tripeptide 7 | C_16_H_20_N_4_O_4_ | 332,1485 | 6,080 | 4 | HILIC-LC-QTOF-MS | ESI- | 3 | 2,54 | [M-H]^-^ | 0,8 | 0.036ꝉ | 1,433 |
| Tripeptide 8 | C_19_H_26_N_4_O_8_ | 438,1751 | 8,520 | 1 | HILIC-LC-QTOF-MS | ESI- | 4 | 6,26 | [M-H+CH3COO]^-^ | 1,2 | 0,027 | 1,315 |
| Tripeptide 9 | C_18_H_18_N_2_O_8_ | 390,1063 | 13,570 | 2 | HILIC-LC-QTOF-MS | ESI- | 4 | 3,39 | [M+Cl]^-^ | 0,8 | 0.037ꝉ | 1,431 |
| Tripeptide 10 | C_13_H_24_N_4_O_5_S | 348,1467 | 13,580 | 7 | HILIC-LC-QTOF-MS | ESI- | 4 | 4,32 | [M+Cl]^-^ | 0,9 | 0,014 | 1,372 |
| Tripeptide 11 | C_19_H_26_N_4_O_4_S_2_ | 438,1395 | 13,740 | 1 | HILIC-LC-QTOF-MS | ESI- | 4 | 3,24 | [M-H+CH3COO]^-^ | 1,3 | 0.003ꝉ | 1,515 |
| Tripeptide 14 | C_21_H_26_N_6_O_5_ | 442,1965 | 3,690 | 0 | RP-LC-QTOF-MS | ESI+ | 4 | 7,31 | [M+H]^+^ | 1,5 | 0,021 | 1,300 |
| ***Benzene and substituted derivatives*** | | | | | | | | | | | | |
| Aminophenol | C_6_H_7_NO | 109,0528 | 1,290 | 6 | RP-LC-QTOF-MS | ESI+ | 3 | 4,28 | [M+H]^+^ | 0,7 | 0.020ꝉ | 1,429 |
| Benzenediol // Pyrocatechol | C_6_H_6_O_2_ | 110,0368 | 1,740 | 5 | HILIC-LC-QTOF-MS | ESI- | 3 | 2,24 | [M-H]^-^ | 0,9 | 0.032ꝉ | 1,443 |
| Benzyl alcohol | C_7_H_8_O | 108,0575 | 8,178 | - | GC-QTOF-MS | ESI+ | 1 | 9,38 | - | 0,4 | - | 1,047 |
| Methyl phenylacetate // Phenylethyl formate | C_9_H_10_O_2_ | 150,0681 | 1,830 | 3 | HILIC-LC-QTOF-MS | ESI- | 3 | 0,82 | [M-H]^-^ | 1,1 | 0.009ꝉ | 1,503 |
| Phenol | C_6_H_6_O | 94,0419 | 2,040 | 5 | HILIC-LC-QTOF-MS | ESI- | 3 | 2,31 | [M-H]^-^ | 0,9 | 0.018ꝉ | 1,478 |
| Phenyllactic acid | C_9_H_10_O_3_ | 166,0630 | 13,978 | - | GC-QTOF-MS | ESI+ | 1 | 13,55 | - | 0,6 | - | 1,268 |
| Syringic acid | C_9_H_10_O_5_ | 198,0528 | 2,440 | 1 | HILIC-LC-QTOF-MS | ESI- | 3 | 1,49 | [M-H]^-^ | 1,1 | 0.014ꝉ | 1,490 |
| Trihydroxydimethoxy methylanthraquinone | C_17_H_14_O_7_ | 330,0740 | 8,100 | 3 | RP-LC-QTOF-MS | ESI+ | 3 | 16,17 | [M+H-H2O]^+^ | 1,6 | 0,030 | 1,262 |
| Tyramine | C_8_H_11_NO | 137,0841 | 11,700 | 4 | RP-LC-QTOF-MS | ESI+ | 4 | 6,41 | [M+H-H2O]^+^ | 0,7 | 0.014ꝉ | 1,447 |
| Vanillic acid | C_8_H_8_O_4_ | 168,0423 | 1,900 | 2 | HILIC-LC-QTOF-MS | ESI- | 3 | 1,96 | [M-H]^-^ | 1,1 | 0,047 | 1,245 |
| Vanillin acetate | C_10_H_10_O_4_ | 194,0579 | 1,510 | 3 | HILIC-LC-QTOF-MS | ESI- | 3 | 3,53 | [M-H]^-^ | 0,9 | 0,039 | 1,270 |
| Verimol H | C_20_H_24_O_4_ | 328,1675 | 17,680 | 4 | RP-LC-QTOF-MS | ESI+ | 3 | 14,79 | [M+H]^+^ | 1,4 | 0,034 | 1,249 |
| ***Carbohydrates and carbohydrate conjugates*** | | | | | | | | | | | | |
| Arabinose | C_5_H_10_O_5_ | 150,0528 | 15,023 | - | GC-QTOF-MS | ESI+ | 1 | 11,72 | - | 1,2 | - | 1,186 |
| Fructose | C_6_H_12_O_6_ | 180,0634 | 17,325 | - | GC-QTOF-MS | ESI+ | 1 | 11,60 | - | 2,0 | 0,013 | 1,769 |
| Galactose | C_6_H_12_O_6_ | 180,0634 | 17,596 | - | GC-QTOF-MS | ESI+ | 1 | 10,09 | - | 1,9 | 0,025 | 1,695 |
| Glucocaffeic acid | C_15_H_18_O_9_ | 342,0951 | 8,220 | 3 | RP-LC-QTOF-MS | ESI+ | 3 | 4,05 | [M+H-H2O]^+^ | 1,3 | 0.050ꝉ | 1,373 |
| Mannitol // Sorbitol | C_6_H_14_O_6_ | 182,0790 | 17.928 // 8.510 | - | GC-QTOF-MS // HILIC-LC-QTOF-MS | ESI± | 1 | 8,29 | [M-H]^-^ | 1,4 | - | 1,367 |
| Methyl galactoside | C_7_H_14_O_6_ | 194,0790 | 20,289 | - | GC-QTOF-MS | ESI+ | 1 | 7,36 | - | 0,7 | - | 1,296 |
| Mucic acid | C_6_H_10_O_8_ | 210,0376 | 18,786 | - | GC-QTOF-MS | ESI+ | 1 | 14,97 | - | 1,5 | 0,012 | 1,774 |
| Maltose // Lactose | C_12_H_22_O_11_ | 342,1162 | 13,490 | 3 | HILIC-LC-QTOF-MS | ESI- | 3 | 0,66 | [M-H]^-^ | 1,3 | 0,026 | 1,320 |
| ***Carboxylic acids and derivatives*** | | | | | | | | | | | | |
| Citric acid | C_6_H_8_O_7_ | 192,0270 | 16,671 | - | GC-QTOF-MS | ESI+ | 1 | 28,77 | - | 1,4 | - | 1,195 |
| L-Aspartic acid | C_4_H_7_NO_4_ | 133,0375 | 11,621 | - | GC-QTOF-MS | ESI+ | 1 | 13,68 | - | 1,1 | - | 1,177 |
| Parapyruvate | C_6_H_8_O_6_ | 176,0321 | 6,480 | 2 | HILIC-LC-QTOF-MS | ESI- | 3 | 1,42 | [M-H]^-^ | 0,9 | 0.017ꝉ | 1,482 |
| ***Cinnamic acids and derivatives*** | | | | | | | | | | | | |
| Caffeic acid // Hydroxycoumarin | C_9_H_8_O_4_ // C_9_H_6_O_3_ | 180.0423 // 162.0317 | 7,890 | 3 // 4 | RP-LC-QTOF-MS | ESI+ | 3 | 13,28 | [M+H-H2O]^+^ // [M+H]^+^ | 0,6 | 0,031 | 1,258 |
| Coumaric acid // 3-Oxo-3-phenylpropanoate | C_9_H_8_O_3_ | 164,0473 | 4,820 | 2 | HILIC-LC-QTOF-MS | ESI- | 3 | 2,26 | [M-H]^-^ | 1,1 | 0,042 | 1,262 |
| Feruloylquinolactone | C_17_H_18_O_8_ | 350,1002 | 1,760 | 2 | HILIC-LC-QTOF-MS | ESI- | 3 | 0,15 | [M-H]^-^ | 0,9 | 0,023 | 1,333 |
| Vinyl caffeate | C_11_H_10_O_4_ | 206,0579 | 1.460 // 7.580 | 1 | HILIC-LC-QTOF-MS // RP-LC-QTOF-MS | ESI- | 2 | 2,72 | [M-H]^-^ // [M+H]^+^ | 0,9 | 0.042ꝉ | 1,422 |
| ***Quinic acids and derivatives*** | | | | | | | | | | | | |
| Coumaroylquinic acid | C_16_H_18_O_8_ | 338,1002 | 10.130 // 4.810 | 4 | RP-LC-QTOF-MS // HILIC-LC-QTOF-MS | ESI± | 3 | 2,09 | [M+H]^+^ // [M-H]^-^ | 1,3 | 0,025 | 1,282 |
| 3,4-Dicaffeoylquinic acid | C_25_H_24_O_12_ | 516,1268 | 13,590 | 4 | RP-LC-QTOF-MS | ESI+ | 1 | 1,93 | [M+H-H2O]^+^ // [M+H]^+^ | 1,2 | 0.013ꝉ | 1,452 |
| ***Organic acids and derivatives*** | | | | | | | | | | | | |
| 2-ketobutyric acid | C_4_H_6_O_3_ | 102,0317 | 7,426 | - | GC-QTOF-MS | ESI+ | 1 | 25,06 | - | 1,2 | - | 1,380 |
| 3-Hydroxybutyric acid | C_4_H_8_O_3_ | 104,0473 | 8,279 | - | GC-QTOF-MS | ESI+ | 1 | 5,82 | - | 0,3 | 0,005 | 1,840 |
| Alpha Ketoglutaric acid | C_5_H_6_O_5_ | 146,0215 | 13,848 | - | GC-QTOF-MS | ESI+ | 1 | 20,51 | - | 0,5 | - | 1,275 |
| Hydroxyindolelactate | C_11_H_11_NO_4_ | 221,0688 | 6,660 | 2 | HILIC-LC-QTOF-MS | ESI- | 3 | 1,40 | [M-H]^-^ | 1,2 | 0,009 | 1,402 |
| Isopropyl formate | C_4_H_8_O_2_ | 88,0524 | 4,340 | 3 | HILIC-LC-QTOF-MS | ESI- | 3 | 1,78 | [M-H]^-^ | 0,0 | 0.003ꝉ | 1,515 |
| Pyruvic acid | C_3_H_4_O_3_ | 88,0160 | 6,622 | - | GC-QTOF-MS | ESI+ | 1 | 5,88 | - | 1,5 | 0,012 | 1,774 |
| ***Fatty Acyls*** | | | | | | | | | | | | |
| Arachidonoyl Serotonin | C_30_H_42_N_2_O_2_ | 462,3246 | 6,780 | 5 | HILIC-LC-QTOF-MS | ESI- | 4 | 1,53 | [M+Cl]^-^ | 0,8 | 0.022ꝉ | 1,467 |
| Dodecanoic acid | C_12_H_24_O_2_ | 200,1776 | 14,629 | - | GC-QTOF-MS | ESI+ | 1 | 10,09 | - | 1,3 | - | 1,267 |
| Formylglutarate | C_6_H_8_O_5_ | 160,0372 | 6,420 | 3 | RP-LC-QTOF-MS | ESI+ | 3 | 3,46 | [M+H-H2O]^+^ | 1,3 | 0.013ꝉ | 1,451 |
| Norlinolenic acid | C_17_H_28_O_2_ | 264,2089 | 19,500 | 4 | RP-LC-QTOF-MS | ESI+ | 3 | 10,34 | [M+H-H2O]^+^ | ↑ | 0.035ꝉ | 1,400 |
| Palmitic acid | C_16_H_32_O_2_ | 256,2402 | 18,698 | - | GC-QTOF-MS | ESI+ | 1 | 5,15 | - | 0,7 | - | 1,436 |
| ***Glycerophospholipids*** | | | | | | | | | | | | |
| PA 36:2 | C_39_H_73_O_8_P | 700,5043 | 4,360 | 3 | HILIC-LC-QTOF-MS | ESI- | 3 | 2,37 | [M-H]^-^ | 0,4 | 0.022ꝉ | 1,465 |
| PG 33:5 | C_39_H_67_O_10_P | 726,4472 | 7,070 | 6 | HILIC-LC-QTOF-MS | ESI- | 3 | 0,64 | [M-H]^-^ | 0,8 | 0.009ꝉ | 1,501 |
| ***Organoheterocyclic compounds*** | | | | | | | | | | | | |
| Butyrolactone | C_4_H_6_O_2_ | 86,0368 | 2,330 | 4 | HILIC-LC-QTOF-MS | ESI- | 4 | 1,48 | [M-H]^-^ | 0,9 | 0.025ꝉ | 1,461 |
| Caffeine | C_8_H_10_N_4_O_2_ | 194,0804 | 16,885 | - | GC-QTOF-MS | ESI+ | 1 | 7,43 | - | 1,3 | - | 1,014 |
| Furfuryl acetate | C_7_H_8_O_3_ | 140,0473 | 6,680 | 5 | RP-LC-QTOF-MS | ESI+ | 2 | 4,04 | M+H | 0,0 | 0.004ꝉ | 1,465 |
| Lipoamide | C_8_H_15_NOS_2_ | 205,0595 | 15,580 | 6 | HILIC-LC-QTOF-MS | ESI- | 3 | 1,70 | [M-H]^-^ | 0,7 | 0,016 | 1,363 |
| Methoxymethyl furan // Methyl cyclopentanedione | C_6_H_8_O_2_ | 112,0524 | 2,040 | 4 | HILIC-LC-QTOF-MS | ESI- | 3 | 2,62 | [M-H]^-^ | 0,9 | 0.019ꝉ | 1,473 |
| Mozambioside // Cafamarine | C_26_H_36_O_10_ | 508,2308 | 6.190 // 12.690 | 3 | HILIC-LC-QTOF-MS // RP-LC-QTOF-MS | ESI- | 3 | 2,37 | [M-H]^-^ // [M+H]^+^ // [M+Na]^+^ | 1,4 | 0.035ꝉ | 1,436 |
| Riboflavin | C_17_H_20_N_4_O_6_ | 376,1383 | 16,501 | - | GC-QTOF-MS | ESI+ | 1 | 15,24 | - | 0,8 | - | 1,164 |
| ***Organooxygen compounds*** | | | | | | | | | | | | |
| 1,3-propanediol | C_3_H_8_O_2_ | 76,0524 | 6,714 | - | GC-QTOF-MS | ESI+ | 1 | 8,00 | - | 3,1 | 0,001 | 1,890 |
| 2,3-butanediol | C_4_H_10_O_2_ | 90,0681 | 6,651 | - | GC-QTOF-MS | ESI+ | 1 | 5,65 | - | 1,5 | 0,010 | 1,791 |
| 5-Hydroxymethylfurfural | C_6_H_6_O_3_ | 126,0317 | 11,623 | - | GC-QTOF-MS | ESI+ | 1 | 9,47 | - | 1,1 | - | 1,177 |
| Ethyl cyclopentanedione //Dimethyl cyclopentanedione // Methyl cyclohexanedione | C_7_H_10_O_2_ | 126,0681 | 6,330 | 4 | HILIC-LC-QTOF-MS | ESI- | 3 | 2,21 | [M-H]^-^ | 0,9 | 0.045ꝉ | 1,413 |
| Furanylbutanone | C_8_H_10_O_2_ | 138,0681 | 4,790 | 4 | HILIC-LC-QTOF-MS | ESI- | 4 | 1,59 | [M-H]^-^ | 1,1 | 0,026 | 1,317 |
| Furyl methyl diketone | C_7_H_6_O_3_ | 138,0317 | 4,400 | 3 | HILIC-LC-QTOF-MS | ESI- | 3 | 3,92 | [M-H]^-^ | 0,9 | 0,023 | 1,332 |
| Hydroxyacetophenone | C_8_H_8_O_2_ | 136,0524 | 1,730 | 3 | HILIC-LC-QTOF-MS | ESI- | 2 | 3,19 | [M-H]^-^ | 1,1 | 0.021ꝉ | 1,469 |
| Pyrrole carboxaldehyde | C_5_H_5_NO | 95,0371 | 6,090 | 5 | HILIC-LC-QTOF-MS | ESI- | 3 | 3,00 | [M-H]^-^ | 0,9 | 0.031ꝉ | 1,447 |
| Shikimic acid | C_7_H_10_O_5_ | 174,0528 | 2,220 | 1 | HILIC-LC-QTOF-MS | ESI- | 3 | 1,53 | [M+Cl]^-^ // [M-H]^-^ // [M-H-H2O]^-^ | 0,9 | 0.035ꝉ | 1,435 |
| ***Phenylpropanoids and polyketides*** | | | | | | | | | | | | |
| Ethyl vanillin //Antiarol | C_9_H_10_O_3_ // C_9_H_12_O_4_ | 166.0630 // 184.07360 | 5,630 | 2 // 1 | HILIC-LC-QTOF-MS | ESI- | 3 | 2,03 | [M-H]^-^ // [M-H-H2O]^-^ | 0,7 | 0.003ꝉ | 1,515 |
| ***Prenol Lipids*** | | | | | | | | | | | | |
| Genipin gentiobioside | C_23_H_34_O_15_ | 550,1898 | 8,490 | 3 | HILIC-LC-QTOF-MS | ESI- | 3 | 3,61 | [M-H]^-^ | 1,2 | 0.014ꝉ | 1,488 |
| ***Purines and purine derivatives*** | | | | | | | | | | | | |
| Adenine | C_5_H_5_N_5_ | 135,0545 | 5,940 | 4 | HILIC-LC-QTOF-MS | ESI- | 3 | 1,19 | [M-H]^-^ | 0,9 | 0.025ꝉ | 1,461 |
| Theobromine | C_7_H_8_N_4_O_2_ | 180,0647 | 5,950 | 3 | RP-LC-QTOF-MS | ESI+ | 2 | 9,91 | [M+H]^+^ | 1,4 | 0,009 | 1,364 |
| ***Pyridines and derivatives*** | | | | | | | | | | | | |
| Ethyl methylpyridine | C_8_H_11_N | 121,0891 | 2,420 | 5 | RP-LC-QTOF-MS | ESI+ | 3 | 1,70 | [M+H]^+^ | 0,7 | 0.020ꝉ | 1,429 |
| Ethyl pyridine | C_7_H_9_N | 107,0735 | 1,620 | 4 | RP-LC-QTOF-MS | ESI+ | 2 | 6,92 | [M+H]^+^ | 0,8 | 0.028ꝉ | 1,415 |

^a^CV for QC, coefficient of variation in the metabolites in the QC samples; ^b^p-value corresponding to the p-values calculated by Tukey pairwise comparisons (p-value < 0.05) and ꝉ corresponding to the p-values calculated by the Benjamini–Hochberg false discovery rate post hoc correction (FDR < 0.05); ^c^VIP, variable importance in projection; Fold Change, indicate the change in the abundance of the specified comparison calculated as (average case/average control), where values greater than 1 indicated an increase while values less than 1 indicate a decrease in the case group; RT: Retention time; RP-LC-QTOF-MS: liquid chromatography-quadrupole time-of-flight mass spectrometry; HILIC-LC-MS-QTOF: Hydrophilic Interaction Chromatography system coupled to a Q-TOF Mass Spectrometer; GC-QTOF-MS: Gas Chromatography coupled to a Q-TOF Mass Spectrometer. The ID levels show confidence in the identification and were assigned from 0 to 4, where: 0 = unambiguous 3D structure; 1 = confident 2D structure or reference standard match; 2 = probable structure or coincidence with literature spectra; 3 = possible structure, confirmation with molecular formula; and 4 = Unknown feature or only match with libraries.
